# Supplementary material for: Mus81-Mms4 endonuclease is an Esc2-STUbL-Cullin8 mitotic substrate impacting on genome integrity
Source: Nat Commun. 2020 Nov 12;11:5746. doi: 10.1038/s41467-020-19503-4 (PMC7665200; doi:10.1038/s41467-020-19503-4)
Supplement: Supplementary file 1 — Supplementary Information [file 41467_2020_19503_MOESM1_ESM.pdf]

SUPPLEMENTARY INFORMATION

Mus81-Mms4 endonuclease is an Esc2-STUbL-Cullin8 mitotic substrate impacting on genome integrity

Anja Waizenegger, Madhusoodanan Urulangodi, Carl P. Lehmann, Teresa Anne Clarisse Reyes, Irene Saugar, Jose Antonio Tercero, Barnabas Szakal, and Dana Branzei*

* Corresponding author

E-mail: dana.branzei@ifom.eu (D.B.)

SUPPLEMENTARY FIGURES AND FIGURE LEGENDS

Supplementary Fig. 1

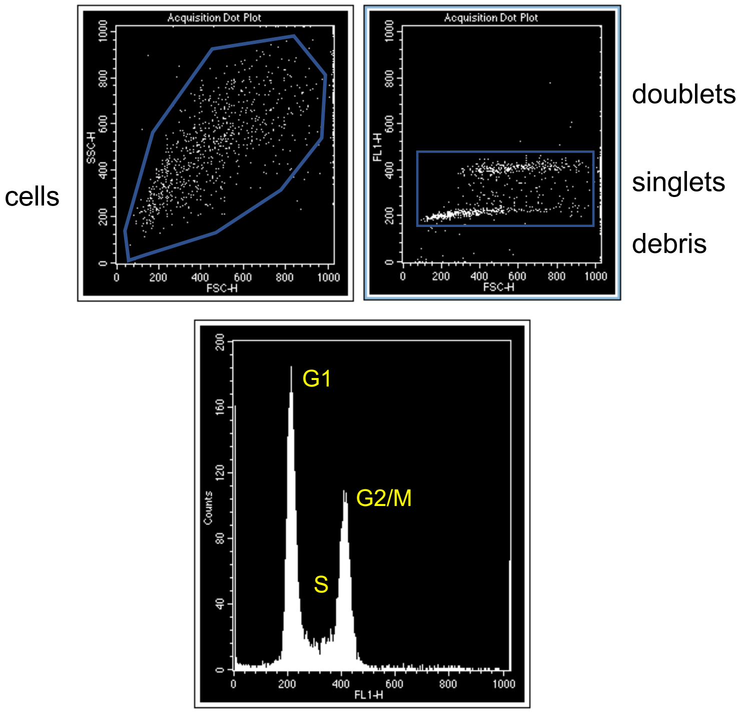

Supplementary Fig. 1. Flow Cytometry gating strategy and representative image

For all experiments in which cell cycle progression was followed, samples were gated on SSC-H and FSC-H as well as on FL1-H and FSC-H to exclude doublets and debris. Then a histogram of FL1-H values was generated from the remaining cells. A value of 200 in FL1-H represents the G1 population with a 1N DNA content and a value of 400 represents the G2 population with a 2N DNA content. 50.000 cells per sample were analysed.

Supplementary Fig. 2

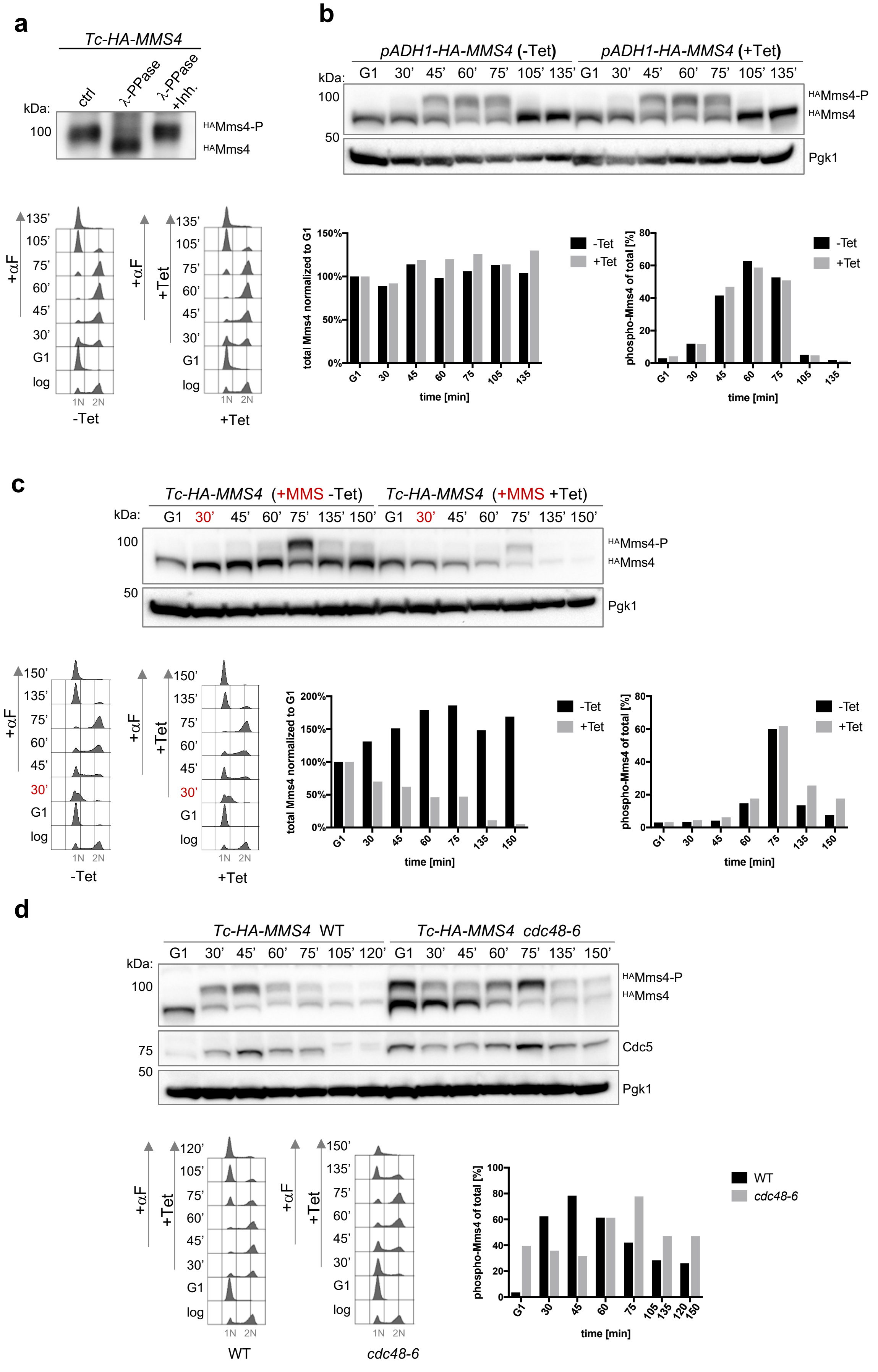

Supplementary Fig. 2. Mms4 is targeted for proteasome-dependent turnover in mitosis.

(a) Immunoprecipitated HA-Mms4 from G2/M arrested Tc-HA-Mms4 (WT) cells was either mock treated (ctrl), treated with lambda-phosphatase (λ-PPase), or with lambda-phosphatase and phosphatase inhibitors (λ-PPase+Inh.). Samples were analyzed by Western Blot using an anti-HA antibody. **(b)** Time course experiment analyzing Mms4 species and levels within one cell cycle in a control strain lacking the Tc-element. Logarithmically (log) grown *pADH1-HA-MMS4* cells were synchronized in G1 phase with α-factor (αF), then released in YPD medium in the absence (-Tet) or presence of 1 mM Tetracycline (+Tet). After cells reached G2/M, α-factor was again added to the culture. Samples were taken at the indicated timepoints and the presence of HA-tagged Mms4 was analyzed by Western Blot. Pgk1 served as loading control. Total levels of Mms4 were quantified by normalization to the loading control and shown relative to the G1 phase sample. Additionally, the percentage of phosphorylated Mms4 was plotted. Cell cycle progression of the cells during the experiment was followed by flow cytometry analysis. 1N and 2N indicate G1 and G2/M phase respectively. **(c)** Time course experiment analyzing Mms4 species and levels within one cell cycle following acute treatment with MMS. Logarithmically grown *Tc-HA-MMS4* cells were synchronized in G1 phase with α-factor (αF), then released in MMS-containing media (YPD+0.01%MMS) for 30 min (highlighted in red) in the absence (-Tet) or presence of Tetracycline (+Tet). Subsequently, cells were washed with YP and released in YPD in the absence (-Tet) or presence of Tetracycline (+Tet) for the remaining time of the experiment. After cells reached G2/M, α-factor was again added to the culture. Samples were taken at the indicated timepoints and the presence of HA-tagged Mms4 species was analyzed as in **(b)**. **(d)** Time course experiment analyzing Mms4 species within one cell cycle in WT and *cdc48-6* cells. Logarithmically grown *Tc-HA-MMS4* cells of the indicated genotype were synchronized in G1 phase with α-factor (αF) and released in YPD medium containing Tetracycline (Tet) at 30°C. After cells reached G2/M, α-factor was again added to the culture. Samples were taken at the indicated timepoints and analyzed as in **(b)**. Source data are provided as a Source Data file.

Supplementary Fig. 3

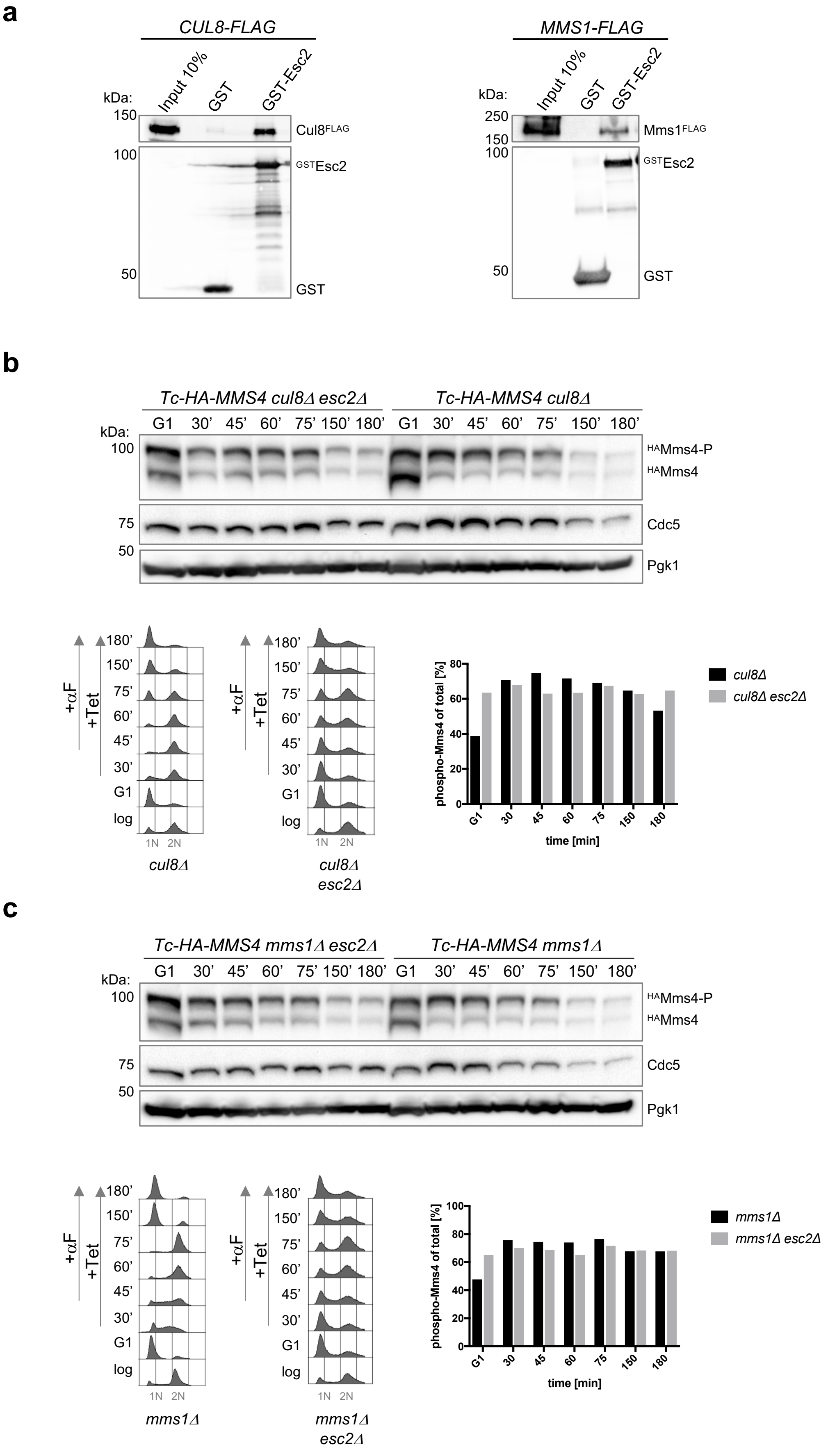

Supplementary Fig. 3. Cullin 8 complex interacts with Esc2 and together regulate Mms4-P abundance. (a) *In vivo* pulldown assays of GST-Esc2 with endogenous Cul8 or Mms1. GST-Esc2 protein was expressed and purified from *E. coli* and used for interaction studies. Yeast total cell lysates were prepared from cells expressing *CUL8-FLAG* or *MMS1-FLAG* and incubated with GST or GST-Esc2. The protein complexes formed on Glutathione sepharose beads were analyzed by Western Blot using anti-FLAG and anti-GST antibodies. **(b)** Time course experiment analyzing the Mms4 species and levels within one cell cycle in *cul8Δ esc2Δ* cells versus *cul8Δ*. Logarithmically (log) grown cells of the indicated genotype expressing *Tc-HA-MMS4* were synchronized in G1 phase with α-factor (αF) and then released in YPD medium containing 1 mM Tetracycline (Tet). After cells reached G2/M, α-factor was again added to the culture to arrest cells in the next G1 phase. Samples were taken at the indicated timepoints and the presence of HA-tagged unphosphorylated (^{HA}Mms4) or phosphorylated Mms4 (^{HA}Mms4-P) was analyzed by Western Blot. Pgk1 served as loading control. The percentage of phosphorylated Mms4 compared to total levels of Mms4 was quantified and plotted. Cell cycle progression of the cells during the experiment was followed by flow cytometry analysis. 1N and 2N indicate G1 and G2/M phase respectively. Cdc5 levels were detected using an anti-Cdc5 antibody. **(c)** Time course experiment analyzing the Mms4 species and levels within one cell cycle in *mms1Δ esc2Δ* cells versus *mms1Δ*. Experimental setup as described in **(b)**. Source data are provided as a Source Data file.

Supplementary Fig. 4

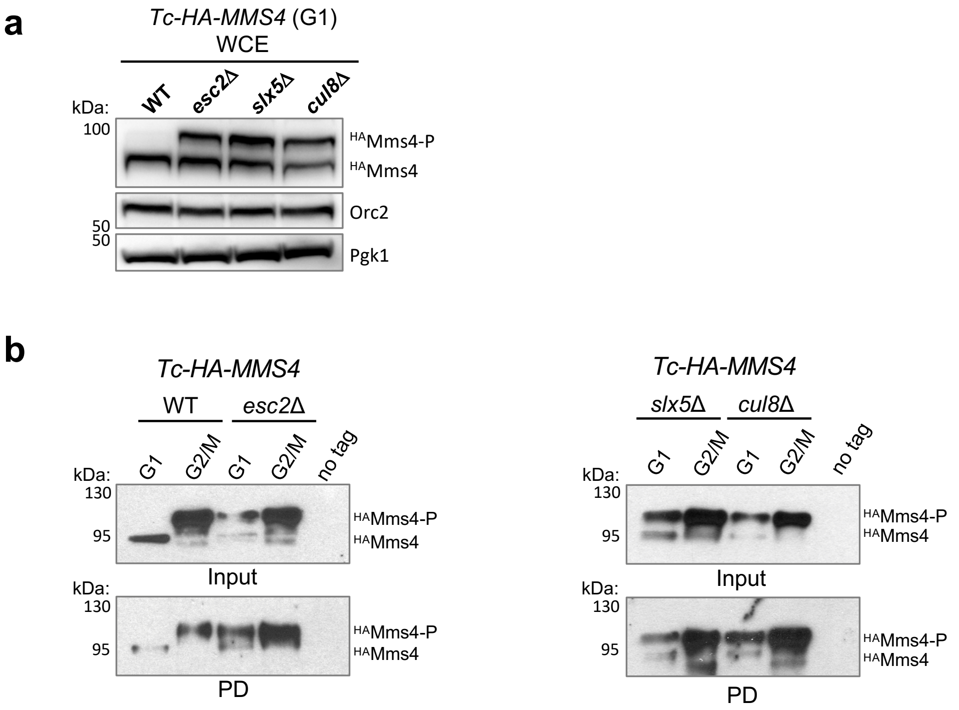

Supplementary Fig. 4. Mms4 levels and species in Esc2, Slx5 and Cul8 defective cells in whole cell extracts and pull downs. (a) Whole cell extracts for chromatin binding assay of HA-Mms4. Western Blot analysis of HA-tagged Mms4 shows Mms4 protein levels of the indicated strains arrested in G1 with α factor in whole cell extracts (WCE). **(b)** Immunoblot for nuclease activity assays of HA-Mms4 in different mutants as shown in Figure 4b. Western Blot analysis of HA-tagged Mms4 shows pulldown efficiency for the indicated strains for G1 and G2/M extracts. PD stands for pulldown. Source data are provided as a Source Data file.

Supplementary Fig. 5

a

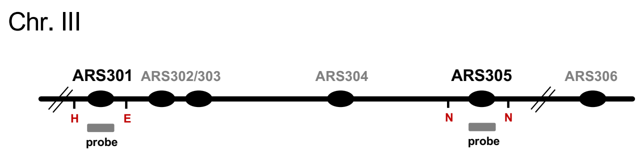

b

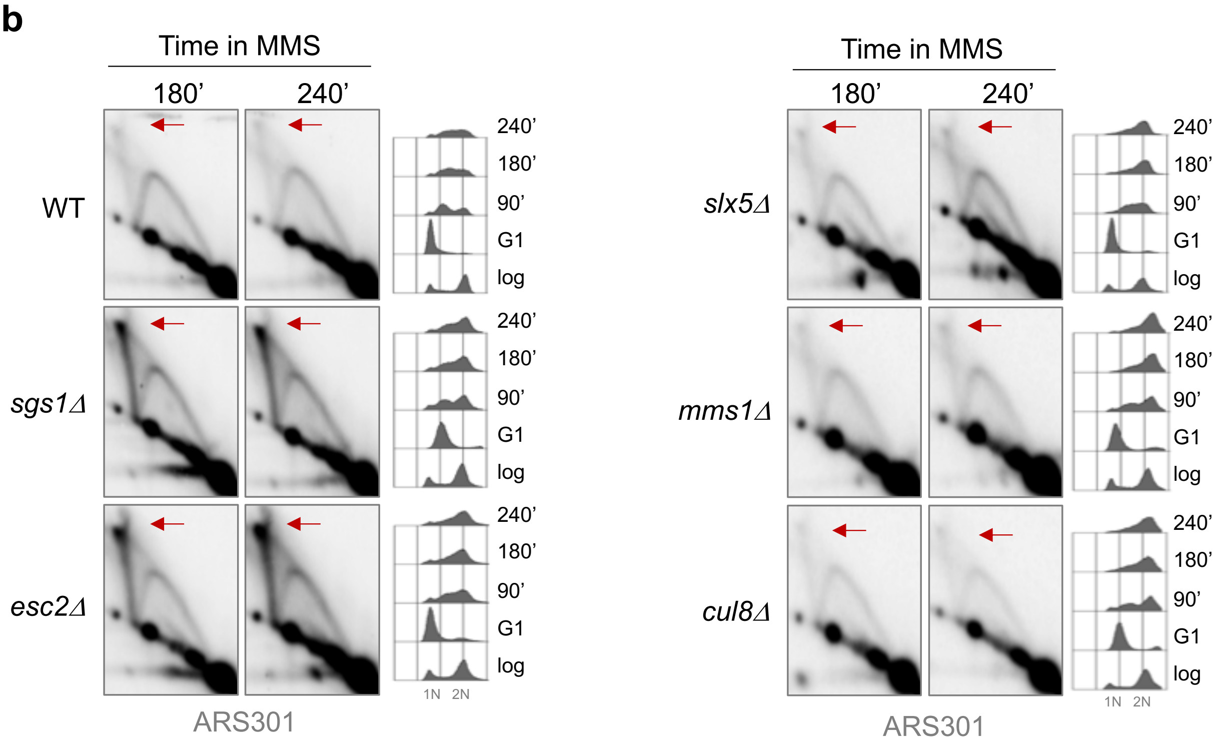

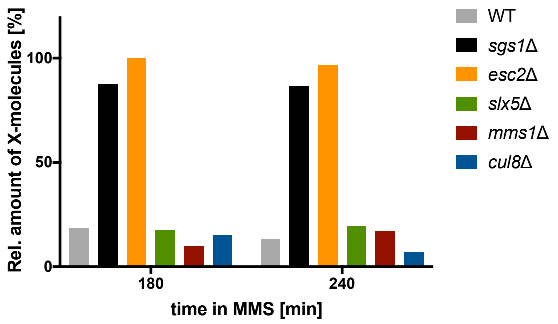

Supplementary Fig. 5. Schematic representation of 2D gel replication intermediates and accumulation of recombination intermediates in *sgs1*Δ and *esc2*Δ mutants, but not in *slx5*Δ, *cul8*Δ and *mms1*Δ. **(a)** Schematic representation of the genomic region on chromosome III containing the late origin of replication ARS301 and the early origin of replication ARS305. The probe for ARS305 spans from 39026 to 41647, the probe for ARS301 from 10135 to 11416. E, H, N indicate the restriction sites for the restriction enzymes *Eco*RV, *Hind*III and *Nco*I. **(b)** 2D gel profiles of replication intermediates isolated from cells of the indicated genotype. Wildtype (WT) cells or single deletion strains (*sgs1*Δ, *esc2*Δ, *slx5*Δ, *mms1*Δ, *cul8*Δ) were synchronized in G1 phase with α-factor and then released in medium containing 0.033% MMS. Samples for 2D gel analysis were collected at the indicated time points and cell cycle progression during the experiment was followed by flow cytometry analysis. 1N and 2N indicate G1 and G2/M phase respectively. Replication intermediates were digested with *Eco*RV and *Hind*III and visualized using a radioactively labelled probe specific for ARS301 in 2D gel electrophoresis. The levels of X-molecules, indicated by red arrows, for each strain and timepoint were normalized to the monomer spot and shown relative to the highest value. Source data are provided as a Source Data file.

Supplementary Fig. 6

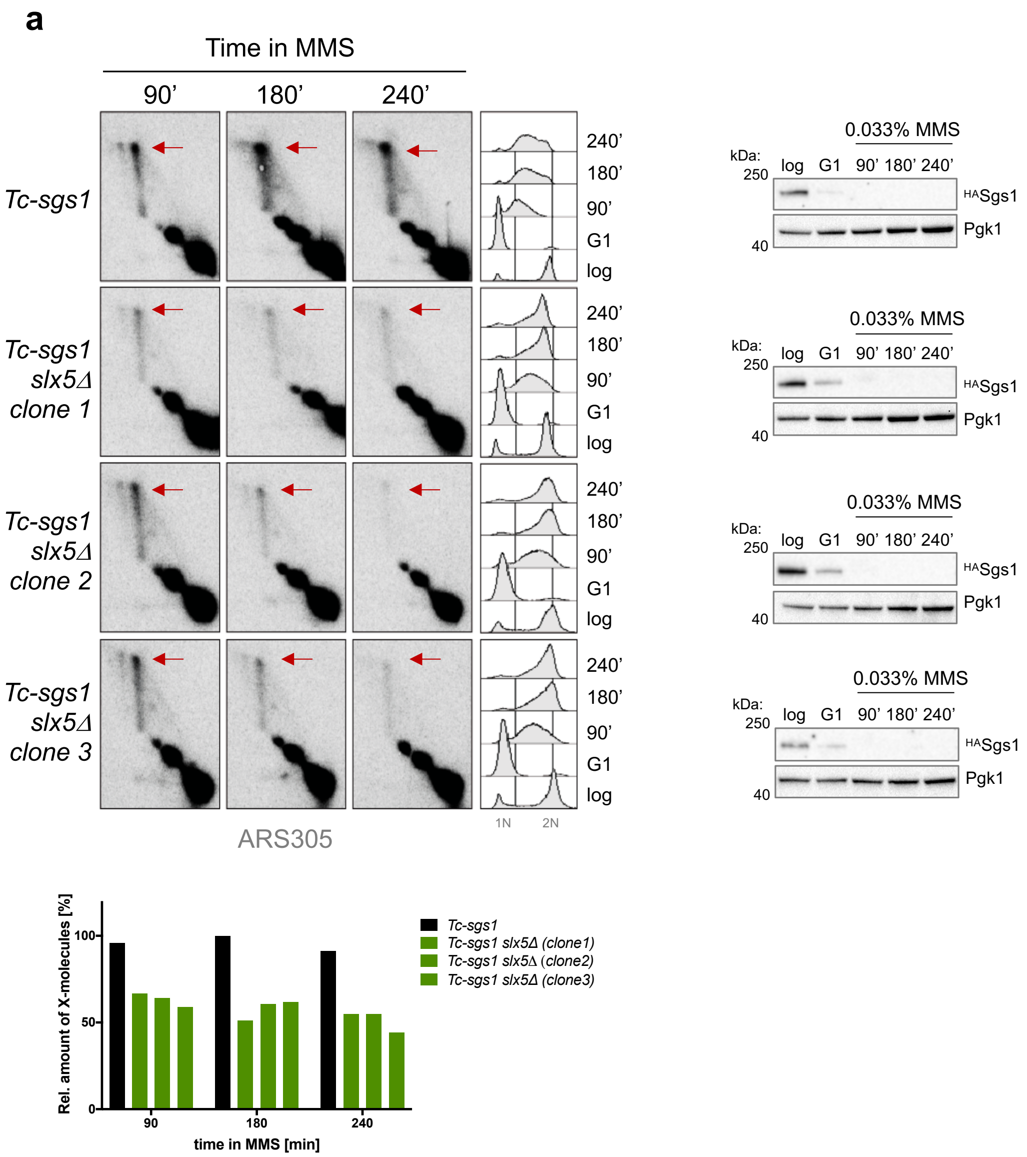

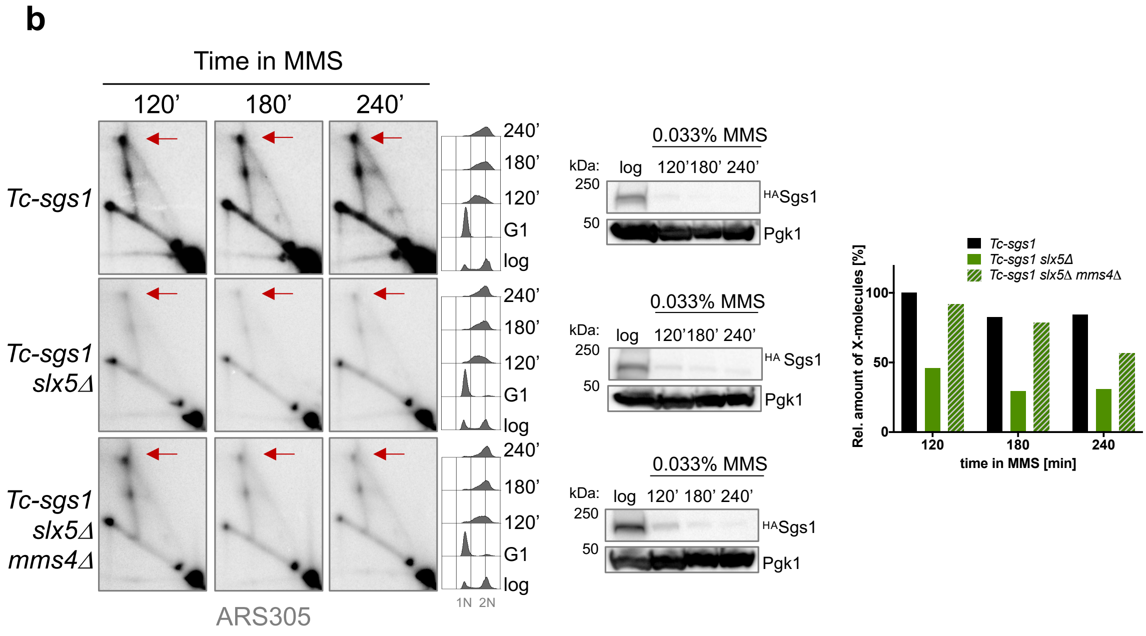

Supplementary Fig. 6. Slx5 protects replication-associated recombination structures by preventing their unscheduled Mms4-Mus81-dependent processing. (a) 2D gel profiles of recombination intermediates isolated from cells of the indicated genotype (3 different clones of *Tc-sgs1 slx5*Δ clones were used). Cells were synchronized in G1 phase with α-factor and then released in medium containing 0.033% MMS. 1 mM Tetracycline was added during G1 arrest and release to cause reduction in Tc-HA-Sgs1 levels. Samples for 2D gel analysis were collected at the indicated time points and levels of HA-tagged Sgs1 were monitored by Western Blot. Pgk1 served as loading control. Cell cycle progression of the cells during the experiment was followed by flow cytometry analysis. 1N and 2N indicate G1 and G2/M phase respectively. Replication intermediates were digested with *EcoR*V and *Hind*III and visualized using a radioactively labelled probe specific for ARS305 in 2D gel electrophoresis. Arrows indicate X-shaped replication intermediates, the levels of which were normalized to the monomer spot and shown relative to the highest value. **(b)** 2D gel profiles of recombination intermediates isolated from cells of the indicated genotype using the same experimental setup as in **(a)**, but replication intermediates were digested with *Nco*I. Source data are provided as a Source Data file.

Supplementary Fig. 7

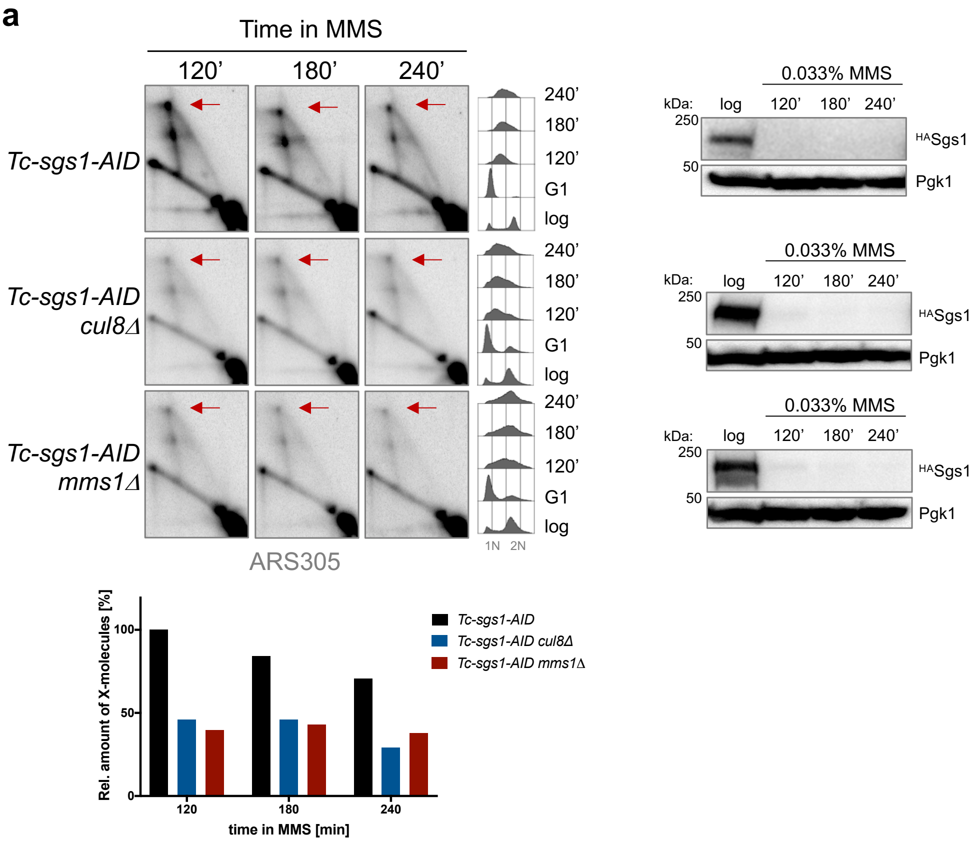

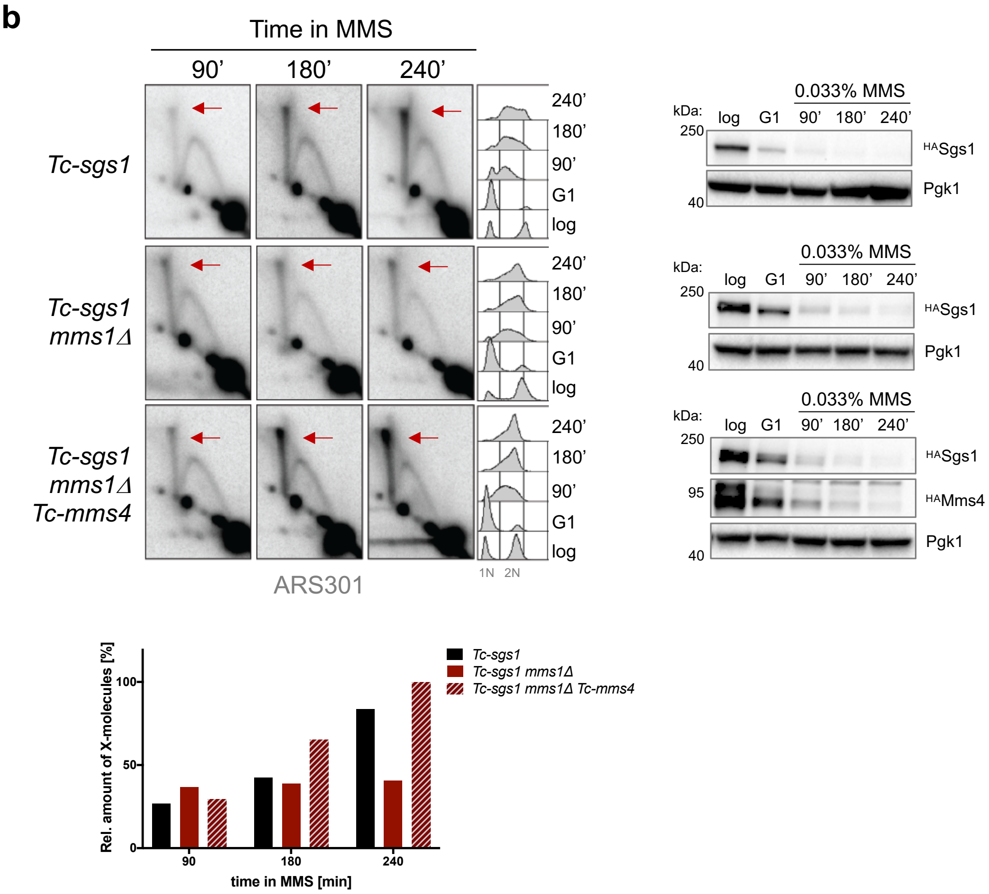

Supplementary Fig. 7. Cul8 and Mms1 protect replication-associated recombination structures by preventing their unscheduled Mms4-Mus81-dependent processing. (a) 2D gel profiles of recombination intermediates isolated from cells of the indicated genotype. Cells were synchronized in G1 phase with α-factor and then released in medium containing 0.033% MMS. 1 mM Tetracycline and 1 mM of Auxin was added during G1 arrest and release to cause depletion in Tc-HA-Sgs1-AID. Samples for 2D gel analysis were collected at the indicated time points and levels of HA-tagged Sgs1 were monitored by Western Blot. Immunodetection of Pgk1 served as a loading control. Cell cycle progression of the cells during the experiment was followed by flow cytometry analysis. 1N and 2N indicate G1 and G2/M phase respectively. Replication intermediates were digested with *Nco*I and visualized using a radioactively labelled probe specific for ARS305 in 2D gel electrophoresis. Arrows indicate X-shaped replication intermediates, the levels of which were normalized to the monomer spot and shown relative to the highest value. **(b)** 2D gel profiles of recombination intermediates isolated from cells of the indicated genotype using a similar experimental setup as in **(a)**, except that only 1 mM Tetracycline was used to cause depletion of Tc-HA-Sgs1 and Tc-HA-Mms4. Moreover, replication intermediates were digested with *EcoR*V and *Hind*III and visualized using a radioactively labelled probe specific for ARS301. Source data are provided as a Source Data file.

Supplementary Fig. 8

a

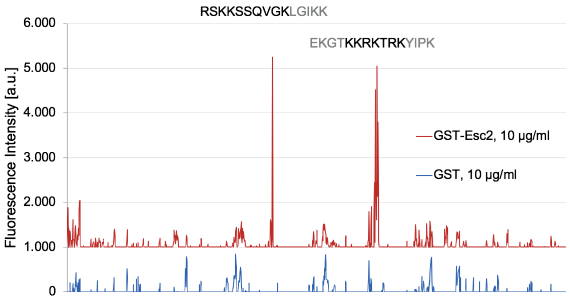

Mms4: peptide 541-555
RSKKSSQVGKLGIKK

Mus81: peptide 121-135
EKGTKKRKTRKYIPK

b

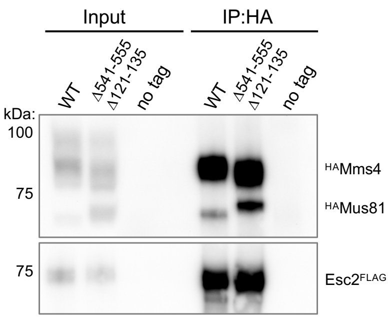

c

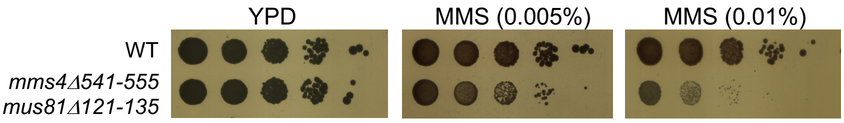

d

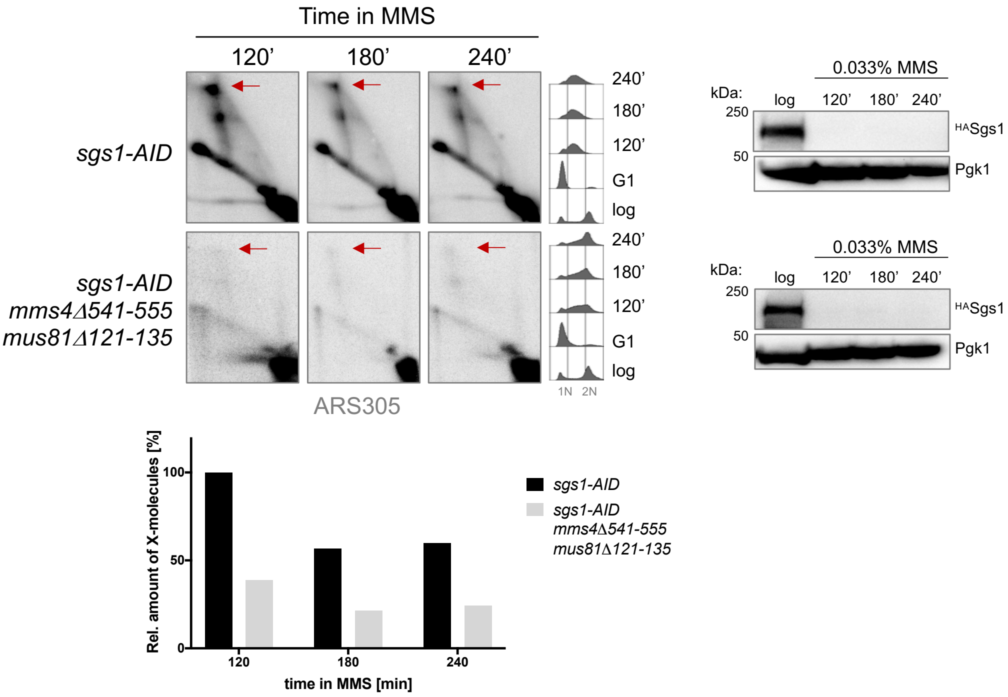

Supplementary Fig. 8. Mapping of peptides within Mms4 and Mus81 engaged in interaction with Esc2 and phenotypes of the internal truncation mutant in regard to damage sensitivity and replication intermediate fragility. (a) Identification of peptides within Mms4 and Mus81 engaged in interaction with GST-Esc2. 15 amino acid (aa) Mms4- and Mus81-derived peptides with peptide-peptide overlaps of 14 aa were used together with purified GST-Esc2 or GST using peptide microarray scan (PEPperMAP protein interaction mappings, PEPperPRINT®). The assay revealed 15 aa peptides RSKKSSQVGKLGIKK at position 541-555 within the Mms4 protein and EKGTKKRKTRKYIPK at position 121-135 within the Mus81 protein as highly specific. **(b)** Co-immunoprecipitation of Mms4-Mus81 with Esc2 in WT and the *mms4Δ541-555 mus81Δ121-135* truncation mutant. HA-tagged Mms4 and HA-tagged Mus81 variants from either WT cells or double truncation mutant *mms4Δ541-555 mus81Δ121-135* were immunoprecipitated using agarose beads coupled to an HA-antibody. Detection of endogenous, C-terminally FLAG-tagged Esc2 and HA-tagged Mms4, Mus81 was carried out by Western Blot analysis using anti-HA and anti-FLAG antibodies. As control, cells without any tag were used. Source data are provided as a Source Data file. **(c)** MMS-sensitivity assay of the *mms4Δ541-555 mus81Δ121-135* truncation mutant. WT cells or *mms4Δ541-555 mus81Δ121-135* cells were spotted in a serial dilution on YPD plates or plates containing the indicated concentration of MMS and incubated at 28ºC for 3 days. **(d)** 2D gel profiles of recombination intermediates isolated from cells of the indicated relevant genotype. Note that both *sgs1-AID* and *mms4Δ541-555 mus81Δ121-135* are expressed from a *Tc-pADH1-HA* promoter. Cells were synchronized in G1 phase with α-factor and then released in medium containing 0.033% MMS. 1 mM Auxin was added during G1 arrest and release. Samples for 2D gel analysis were collected at the indicated time points and levels of HA-tagged Sgs1 were monitored by Western Blot. Pgk1 served as loading control. Cell cycle progression of the cells during the experiment was followed by flow cytometry analysis. 1N and 2N indicate G1 and G2/M phase respectively. Replication intermediates were digested with *Nco*I and visualized using a radioactively labelled probe specific for ARS305 in 2D gel electrophoresis. Signal intensities were quantified, normalized to the monomer spot and shown relative to the highest value. Source data are provided as a Source Data file.

SUPPLEMENTARY TABLES

Supplementary Table 1: *S. cerevisiae* strains used in this study

Nr.	Strain	Genotype	Source
FY1000	W303 wildtype	W303 MATa *ade2-1 can1-100 his3-11-15 leu2-3, 112 trp1-1 ura3-1 RAD5+*	Lab collection
HY7390	*Tc-HA-MMS4*	W303 MATa mms4::pADH1-Tc3-3xHA-Mms4::HPHMX	Lab collection
HY6279	*Tc-HA-MMS4 cim3-1*	W303 MATa cim3-1-URA3 mms4::pADH1-Tc3-3xHA-Mms4::HPHMX	This study
HY7544	*Tc-HA-MMS4 esc2Δ*	W303 MATa mms4::pADH1-Tc3-3xHA-Mms4::HPHMX esc2delta::NATMX	This study
HY7643	*Tc-HA-MMS4 slx5Δ*	W303 MATa mms4::pADH1-Tc3-3xHA-Mms4::HPHMX slx5delta::HIS3	This study
HY7641	*Tc-HA-MMS4 cul8Δ*	W303 MATa mms4::pADH1-Tc3-3xHA-Mms4::HPHMX cul8delta::HIS3	This study
HY7639	*Tc-HA-MMS4 mms1Δ*	W303 MATa mms4::pADH1-Tc3-3xHA-Mms4::HPHMX mms1delta::HIS3	This study
HY10761	*Tc-HA-MMS4 cdc48-6*	W303 MATa cdc48-6 mms4::pADH1-Tc3-3xHA-Mms4::HPHMX	This study
HY8550	*MMS4-PK cim3-1 His-Smt3*	W303 MATa cim3-1-URA3 (TRP1)pSmt3-7His-Smt3 Mms4-PK9::HIS3	This study
HY3274	*MMS4-PK*	W303 MATa Mms4-PK9::HIS3	Lab collection
HY8554	*Tc-HA-MMS4 cim3-1 His-SMT3*	W303 MATa cim3-1-URA3 (TRP1)pSmt3-7His-Smt3 mms4::pADH1-Tc3-3xHA-Mms4::HPHMX	This study
HY4181	*RAD9-FLAG WT*	W303 MATa Rad9-10FLAG::KANMX	Lab collection
HY4180	*RAD9-FLAG esc2Δ*	W303 MATa esc2delta::NATMX::Rad9-10FLAG::KANMX	This study
HY4935	*RAD9-FLAG slx5Δ*	W303 MATa slx5delta::HIS3 Rad9-10FLAG::KANMX	This study
HY8951	*RAD9-FLAG cul8Δ*	W303 MATa Rad9-10FLAG::KANMX cul8delta::HIS3	This study

HY4834	*RAD9-FLAG ddc1Δ*	W303 MATa ddc1delta::HIS3 Rad9-10FLAG::KANMX	This study
HY6142	*CUL8-FLAG*	W303 MATa Cul8-10FLAG::KANMX	This study
HY6146	*MMS1-FLAG*	W303 MATa Mms1-10FLAG::KANMX	This study
FY1081	*esc2Δ*	W303 MATa esc2delta::NATMX	Lab collection
HY4187	*cul8Δ*	W303 MATa cul8delta::HIS3	This study
HY1060	*sgs1Δ*	W303 sgs1delta::HIS3	Lab collection
HY2786	*slx5Δ*	W303 slx5delta::HIS3	Lab collection
HY4165	*mms1Δ*	W303 MATa mms1delta::HIS3	This study
HY4018	*Tc-HA-SGS1*	W303 MATa sgs1::pADH1-Tc3-3xHA-Sgs1(NATMX)	Lab collection
HY9490	*Tc-HA-SGS1 mms1Δ*	W303 MATa sgs1::pADH1-Tc3-3xHA-Sgs1(NATMX) mms1delta::HIS3	This study
HY9677	*Tc-HA-SGS1 mms1Δ Tc-HA-MMS4*	W303 MATa mms4::pADH1-Tc3-3xHA-Mms4::HPHMX mms1delta::HIS3 sgs1::pADH1-Tc3-3xHA-Sgs1(NATMX)	This study
HY4959	*Tc-HA-SGS1 slx5Δ*	W303 MATa slx5delta::HIS3 pADH1-Tc3-3xHA-Sgs1::HPHMX	This study
HY4960	*Tc-HA-SGS1 slx5Δ*	W303 MATa slx5delta::HIS3 pADH1-Tc3-3xHA-Sgs1::KANMX	This study
HY4961	*Tc-HA-SGS1 slx5Δ*	W303 MATa slx5delta::HIS3 pADH1-Tc3-3xHA-Sgs1::KANMX	In this study
HY9794	*Tc-HA-MMS4 pdr5Δ*	W303 MATa mms4::pADH1-Tc3-3xHA-Mms4::HPHMX pdr5delta::HIS3	This study
HY10362	*Tc-HA-MMS4 smt3-KRall cim3-1*	W303 mms4::pADH1-Tc3-3xHA-Mms4::HPHMX cim3-1-URA3 Smt3::HIS3 ura3-52::pADH1-7His-Smt3-KRall-tADH(URA3)	This study
HY10361	*Tc-HA-MMS4 smt3-KRall*	W303 mms4::pADH1-Tc3-3xHA-Mms4::HPHMX (TRP1) Smt3::HIS3 ura3-52::pADH1-7His-Smt3-KRall-tADH1(URA3)	This study
HY10816	*pADH1-MMS4*	W303 MATa pADH1-3HA-Mms4 (NATMX)::Mms4	This study
HY7833	*cim3-1 His-SMT3*	W303 MATa cim3-1-URA3 (TRP1) pSmt3-7His-Smt3	Lab collection
HY10760	*Tc-HA-MMS4 cim3-1 pGAL-His-UBI4*	W303 MATa mms4::pADH1-Tc3-3xHA-Mms4::HPHMX pGALS-7His-Ubi4 (KANMX) cim3-1-URA3	This study

HY10806	*Tc-HA-MMS4 cim3-1 pGAL-His-UBI4 esc2Δ*	W303 MATa mms4::pADH1-Tc3-3xHA-Mms4::HPHMX pGALS-7His-Ubi4 (KANMX) cim3-1-URA3 esc2::NATMX	This study
HY10808	*Tc-HA-MMS4 cim3-1 pGAL-His-UBi4 cul8Δ*	W303 MATa mms4::pADH1-Tc3-3xHA-Mms4::HPHMX pGALS-7His-Ubi4(KANMX) cim3-1-URA3 cul8::HIS3	This study
HY10810	*Tc-HA-MMS4 cim3-1 pGAL-His-UBI4 slx5Δ*	W303 MATa mms4::pADH1-Tc3-3xHA-Mms4::HPHMX pGALS-7His-Ubi4 (KANMX) cim3-1-URA3 slx5::HIS3	This study
HY10812	*cim3-1 pGAL-His-UBI4*	W303 MATa pGALS-7His-Ubi4 (KANMX) cim3-1-URA3	This study
HY7906	*Tc-HA-MMS4 cul8Δ esc2Δ*	W303 MATa mms4::pADH1-Tc3-3xHA-Mms4::HPHMX cul8delta::HIS3 esc2delta::NATMX	This study
HY7905	*Tc-HA-MMS4 mms1Δ esc2Δ*	W303 MATa mms4::pADH1-Tc3-3xHA-Mms4::HPHMX mms1delta::HIS3 esc2delta::NATMX	This study
HY10383	*Tc-HA-SGS1 slx5Δ mms4Δ*	W303 MATa pADH1-Tc3-3xHA - Sgs1::KANMX slx5delta::HIS3 mms4delta::HPHMX	This study
HY10389	*Tc-HA-SGS1 mms1Δ mms4Δ*	W303 MATa pADH1-Tc3-3xHA - Sgs1::KANMX mms1delta::HIS3 mms4delta::HPHMX	In this study
HY10735	*Tc-HA-SGS1-AID cul8Δ*	W303 MATa ura3-1::ADH1-OsTIR1-9Myc (URA3), sgs1::pADH1-Tc3-3xHA(HPHMX)-Sgs1-aid(KANMX) cul8delta::HIS3	This study
HY10733	*Tc-HA-SGS1-AID mms1Δ*	W303 MATa ura3-1::ADH1-OsTIR1-9Myc (URA3), sgs1::pADH1-Tc3-3xHA(HPHMX)-Sgs1-aid(KANMX) mms1delta::HIS3	This study
HY9894	*Tc-HA-mms4Δ541-555 Tc-HA-mus81Δ121-135*	W303 MATa mms4::pADH1-Tc3-3xHA-mms4Δ541-555::KANMX pADH1-Tc3-6xHA-mus81Δ121-135::KANMX	This study
HY10803	*Tc-SGS1-AID*	W303 MATa ura3-1::ADH1-OsTIR1-9Myc (URA3), leu2::GPD1-OsTIR::LEU sgs1::pADH1-Tc3-3xHA(HPHMX)-Sgs1-aid(KANMX)	This study
HY10822	*Tc-SGS1-AID*	W303 MATa ura3-1::ADH1-OsTIR1-9Myc (URA3), leu2::GPD1-	This study

	Tc-HA-mms4Δ541-555<br>*Tc-HA-mus81Δ121-135*	OsTIR::LEU sgs1::pADH1-Tc3-3xHA(HPHMX)-Sgs1-aid(KANMX) mms4::pADH1-Tc3-3xHA-Mms4Δ541-555::KANMX pADH1-Tc3-6xHA-Mus81Δ121-135::KANMX	
HY9987	*Tc-HA-MMS4*<br>*Tc-HA-MUS81*<br>*ESC2-FLAG*	W303 MATa mms4::pADH1-Tc3-3xHA-Mms4::HPHMX pADH1-Tc3-6xHA-Mus81::KANMX Esc2-10FLAG::KANMX	This study
HY9931	*Tc-HA-mms4Δ541-555*<br>*Tc-HA-mus81Δ121-135*<br>*ESC2-FLAG*	W303 MATa mms4::pADH1-Tc3-3xHA-Mms4Δ541-555::KANMX pADH1-Tc3-6xHA-Mus81Δ121-135::KANMX Esc2-10FLAG::NATMX	This study

Supplementary Table 2: Oligonucleotides used in this study for probes and substrates

**Oligonucleotide**	**Sequence**
ARS301 fw	CTAAGTACTCTTCGCAGCAGG
ARS301 rv	GACCGGCTTTCCATGCACTCG
ARS305 fw	GTTCCGAAACAGGACACTTAGC
ARS305 rv	ATCCAGGAGGGACTCAATGTAG
RF-1	GACGCTGCCGAATTCTGGCGTTAGGAGATACCGATAAGCTTCGGCTTAAG
RF-2	ATCGATGTCTCTAGACAGCACGAGCCCTAACGCCAGAATTCGGCAGCGTC
RF-4	GCTCGTGCTGTCTAGAGACATCGAT
