# Supplementary material for: Mus81-Mms4 endonuclease is an Esc2-STUbL-Cullin8 mitotic substrate impacting on genome integrity
Source: Nat Commun. 2020 Nov 12;11:5746. doi: 10.1038/s41467-020-19503-4 (PMC7665200; doi:10.1038/s41467-020-19503-4)
Supplement: Supplementary file 3 — Reporting Summary [file 41467_2020_19503_MOESM3_ESM.pdf]

nature research

Corresponding author(s): Dana Branzei

Last updated by author(s): Sep 22, 2020

Reporting Summary

Nature Research wishes to improve the reproducibility of the work that we publish. This form provides structure for consistency and transparency in reporting. For further information on Nature Research policies, see our Editorial Policies and the Editorial Policy Checklist.

Statistics

For all statistical analyses, confirm that the following items are present in the figure legend, table legend, main text, or Methods section.

n/a	Confirmed	
☐	☒	The exact sample size (*n*) for each experimental group/condition, given as a discrete number and unit of measurement
☐	☒	A statement on whether measurements were taken from distinct samples or whether the same sample was measured repeatedly
☐	☒	The statistical test(s) used AND whether they are one- or two-sided<br>*Only common tests should be described solely by name; describe more complex techniques in the Methods section.*
☒	☐	A description of all covariates tested
☐	☒	A description of any assumptions or corrections, such as tests of normality and adjustment for multiple comparisons
☐	☒	A full description of the statistical parameters including central tendency (e.g. means) or other basic estimates (e.g. regression coefficient) AND variation (e.g. standard deviation) or associated estimates of uncertainty (e.g. confidence intervals)
☐	☒	For null hypothesis testing, the test statistic (e.g. *F*, *t*, *r*) with confidence intervals, effect sizes, degrees of freedom and *P* value noted<br>*Give P values as exact values whenever suitable.*
☒	☐	For Bayesian analysis, information on the choice of priors and Markov chain Monte Carlo settings
☒	☐	For hierarchical and complex designs, identification of the appropriate level for tests and full reporting of outcomes
☒	☐	Estimates of effect sizes (e.g. Cohen's *d*, Pearson's *r*), indicating how they were calculated

Our web collection on statistics for biologists contains articles on many of the points above.

Software and code

Policy information about availability of computer code

Data collection	BIORAD Image Lab Version 5.2.1 for Western Blot acquisition<br>Amersham Typhoon Scanner software V1.0 for 2D gel acquisition<br>BD CellQuest Version 3.3 for FACS sample collection
Data analysis	BIORAD Image Lab Version 5.2.1 for quantification of Western Blots<br>ImageJ 1.50i for preparation of 2D gel pictures<br>GE Healthcare ImageQuant TL 8.2 for quantification of 2D gels<br>Licor Image Studio Lite 5.0 and ImageJ 1.50i for quantification of nuclease activity assays<br>BD CellQuest Version 3.3 for FACS analysis<br>Prism 7.0d (Graphpad) for statistical analyses

For manuscripts utilizing custom algorithms or software that are central to the research but not yet described in published literature, software must be made available to editors and reviewers. We strongly encourage code deposition in a community repository (e.g. GitHub). See the Nature Research guidelines for submitting code & software for further information.

Data

Policy information about availability of data

All manuscripts must include a data availability statement. This statement should provide the following information, where applicable:

- Accession codes, unique identifiers, or web links for publicly available datasets
- A list of figures that have associated raw data
- A description of any restrictions on data availability

The authors declare that all data supporting the findings of this study are available within the paper and its supplementary information files. The source data underlying all figures, Figures 1-7 and Supplementary Figs. 1-8, are provided as a Source Data file.

Field-specific reporting

Please select the one below that is the best fit for your research. If you are not sure, read the appropriate sections before making your selection.

☒ Life sciences ☐ Behavioural & social sciences ☐ Ecological, evolutionary & environmental sciences

For a reference copy of the document with all sections, see nature.com/documents/nr-reporting-summary-flat.pdf

Life sciences study design

All studies must disclose on these points even when the disclosure is negative.

Sample size	No sample-size calculation was performed, sample sizes were chosen e.g. as different time points to cover different stages of cell cycle progression.
Data exclusions	No data were excluded
Replication	In general, all experiments were repeated at least 2 times to ensure reproducibility. We have not experienced cases of non-reproducible data in this study. Experiments in which Mms4 levels were quantified (Fig 1, Fig S2, Fig 2, Fig 3, Fig S3, Fig 7) or the nuclease activity was measured (Fig 4b), were repeated at least 2 times to confirm the result. For the figures only one representative Western Blot was selected and quantified, without statistical anayses. For the cell fractionation experiments, as well as experiments in which Rad9 or Rad53 levels were measured (Fig 4a, Fig 6, Fig 7), the sample size was determined as 3 or 4 to allow statistical analyses and one representative Western Blot was selected for the figures. The mean values of 3 or 4 experiments were calculated and shown with corresponding SEM. Statistical analyses were performed using a two-tailed unpaired t-test with Welch's correction. Pull-down experiments (Fig 2, Fig 3, Fig S3, Fig S8) were repeated at least 2 times and one representative Western Blot was selected for the figure. 2D gel experiments were repeated at least 2 times and in some cases additionally different alleles or regions were used to confirm the results.
Randomization	In each experiment the respective genotype or treatment was with the corresponding controls, and the same experiment was repeated independently several times. Therefore, systematic errors connected to a specific genotype or treatment can be excluded.
Blinding	Before each experiment, the yeast strains that were used were given numbers instead of the genotype or condition and the numbers were then connected to the strains only after analysis.

Reporting for specific materials, systems and methods

We require information from authors about some types of materials, experimental systems and methods used in many studies. Here, indicate whether each material, system or method listed is relevant to your study. If you are not sure if a list item applies to your research, read the appropriate section before selecting a response.

Materials & experimental systems

n/a	Involved in the study	
☐	☒	Antibodies
☒	☐	Eukaryotic cell lines
☒	☐	Palaeontology and archaeology
☒	☐	Animals and other organisms
☒	☐	Human research participants
☒	☐	Clinical data
☒	☐	Dual use research of concern

Methods

n/a	Involved in the study	
☒	☐	ChIP-seq
☐	☒	Flow cytometry
☒	☐	MRI-based neuroimaging

Antibodies

Antibodies used	anti-FLAG (mouse monoclonal, clone M2, Sigma; cat. no: F3165) used in Western Blot used in Western Blot 1:3000<br>anti-HA (mouse monoclonal, clone 16B12, Biolegend; cat. no: 901501) used in Western Blot 1:3000

anti-PK (mouse monoclonal, clone SV5-Pk1, AbD Serotech; cat. no: MCA1360) used in Western Blot 1:3000
anti-Smt3 (rabbit polyclonal, clone Y-84, Santa Cruz; cat. no: sc28649) used in Western Blot 1:2000
anti-Ubiquitin (rabbit polyclonal, abcam; cat. no: ab19247) used in Western Blot 1:2000
anti-Cdc5 (goat polyclonal, clone yN-19, Santa Cruz; cat. no: sc6732) used in Western Blot 1:1000
anti-Rad53 (mouse monoclonal, clone EL7, in house) used in Western Blot 1:5
anti-Orc2 (mouse monoclonal, clone SB46, abcam; cat. no: ab31930) used in Western Blot 1:1000
anti-Pgk1 (mouse monoclonal, clone 22C5D8, Novex Life Technologies; cat. no: 459250) used in Western Blot 1:50.000
anti-Tubulin (mouse monoclonal, clone DM1A, Sigma; cat. no: T9026) used in Western Blot 1:7000
anti-GST (rabbit polyclonal, Cell Signaling, cat. no. 2622S) used in Western Blot 1:3000
anti-rabbit IgG HRP-linked (goat, Cell Signaling; cat. no. 7074) used in Western Blot 1:10.000
anti-mouse IgG HRP-linked (goat, Cell Signaling; cat. no. 7076) used in Western Blot 1:10.000
anti-goat IgG HRP-linked (donkey, Santa Cruz; cat. no. sc2020) used in Western Blot 1:5000
anti-GST Dylight 680 (rabbit polyclonal, Rockland; cat. no. 600-444-200) used for peptide microarray 1:2000
anti-HA (12CA5) Dylight 800 (rabbit polyclonal, Rockland; cat. no. 600-445-384) used for peptide microarray 1:2000

Validation

All antibodies in this study were used for Western Blot analysis in S. cerevisiae yeast samples and the bands for the respective proteins corresponded with the expected size. The application (Western Blot) and species (S. cerevisiae) were indicated on the manufacturers websites.
We have validated anti-FLAG, anti-HA, anti-PK and anti-GST antibodies in Western Blot by using an S. cerevisiae yeast strain without any tag to confirm specificity. Antibodies against Smt3 or Ubiquitin were validated by using an S. cerevisiae yeast strain which was not expressing the respective construct. The Cdc5 antibody was validated by its cell cycle-specific expression. The Rad53 antibody was validated in various publications (Fiorani et. al., 2008; Ferrari et. al., 2017). The Orc2 antibody was validated by its absence in the cytosol fraction and the Tubulin antibody by its absence in the chromatin fraction. The Pgk1 antibody is commonly used as a loading control in many publications (Gay et. al., 2018).

Flow Cytometry

Plots

Confirm that:

☒ The axis labels state the marker and fluorochrome used (e.g. CD4-FITC).

☒ The axis scales are clearly visible. Include numbers along axes only for bottom left plot of group (a 'group' is an analysis of identical markers).

☒ All plots are contour plots with outliers or pseudocolor plots.

☒ A numerical value for number of cells or percentage (with statistics) is provided.

Methodology

Sample preparation

For flow cytometry analysis, approximately 1x107 cells for each timepoint were collected and fixed in 70% ethanol. Cells were suspended in 10 mM Tris pH 7.5 buffer, and RNA and proteins were removed by RNase A (0.4 mg/ml) and proteinase K (1 mg/ml) treatment. Subsequently, cells were stained in SYTOX green solution (1µM) and analyzed using a FACSCalibur Flow Cytometer.

Instrument

BD FACSCalibur Flow Cytometer

Software

BD CellQuest Version 3.3

Cell population abundance

No populations were sorted, only cell cycle progression was followed by analyzing 50.000 cells per sample

Gating strategy

Samples were gated on SSC-H and FSC-H as well as on FL1-H and FSC-H to exclude doublets and debris. Then a histogram of FL1-H values was generated from the remaining cells. A value of 200 in FL1-H represents the G1 population with a 1n DNA content and a value of 400 represents the G2 population with a 2n DNA content.

☒ Tick this box to confirm that a figure exemplifying the gating strategy is provided in the Supplementary Information.
